# The Role of Flavonoids in Alleviating Mammary Gland Inflammation: A Review

**DOI:** 10.3390/vetsci13080743

**Published:** 2026-07-26

**Authors:** Abdul Qadeer, Mohamed Tharwat, Ibrahim F. Halawani, Fuad M. Alzahrani, Khalid J. Alzahrani, Fahad A. Alshanbari, Muhammad Zahoor Khan

**Affiliations:** 1 Department of Medical Sciences, Shandong Xiehe University, Jinan 250109, China; qadeerabdul@sdxiehe.edu.cn; 2 Department of Clinical Sciences, College of Veterinary Medicine, Qassim University, P.O. Box 6622, Buraidah 51452, Saudi Arabia; atieh@qu.edu.sa; 3 Department of Clinical Laboratory Sciences, College of Applied Medical Sciences, Taif University, Taif 21974, Saudi Arabia; 4 Department of Medical Biosciences, College of Veterinary Medicine, Qassim University, P.O. Box 6622, Buraidah 51452, Saudi Arabia; 5 College of Agriculture and Biology, Liaocheng University, Liaocheng 252000, China

**Keywords:** mastitis, flavonoids, anti-inflammatory and antioxidant properties, oxidative stress, blood–milk barrier, gut–mammary axis

## Abstract

Mammary gland inflammation, clinically expressed as mastitis in dairy ruminants and lactating women, arises from a self-amplifying loop of pathogen sensing, oxidative stress and innate immune activation, and remains a leading driver of antibiotic use, milk-quality loss and morbidity. Mounting pressure to reduce antimicrobial dependence has refocused attention on flavonoids—structurally diverse plant polyphenols with multi-target bioactivity, derived mainly from in vitro and rodent mastitis models. This review integrates contemporary evidence on the six principal flavonoid subclasses within a unifying molecular framework. Across subclasses, flavonoids converge on shared targets: the TLR4–MyD88–NF-κB axis, MAPK cascades, the Keap1–Nrf2–ARE antioxidant pathway, the NLRP3 inflammasome and tight-junction proteins of the blood–milk barrier. Less canonical mechanisms—m^6^A epitranscriptomic regulation, ferroptosis suppression, AhR signalling, anti-virulence binding to bacterial enzymes such as IGPD, and gut-microbiota-driven remodelling of the gut–mammary axis—expand the pharmacological landscape. We additionally appraise the subclasses comparatively, identifying flavanones and the flavone baicalin as carrying the strongest translational evidence, and examine the conflicting findings, model limitations, and delivery, residue and regulatory barriers that currently separate mechanistic promise from on-farm application. We outline structure–activity considerations and translational priorities, and position flavonoids as mechanism-rich, antibiotic-sparing candidates for the prevention and adjunctive management of mammary gland inflammation in dairy ruminants.

## Simple Summary

Mastitis, or inflammation of the mammary gland, is one of the most common and costly diseases affecting dairy animals worldwide and a major cause of pain in breastfeeding women. It reduces milk yield and quality, harms animal welfare, and drives heavy antibiotic use, accelerating antimicrobial resistance. Safer, natural alternatives are urgently needed. Flavonoids—plant compounds abundant in citrus and other fruits, vegetables, onions, soybeans, licorice, green tea and forage legumes—act on the very processes that fuel this disease: they neutralize harmful reactive oxygen species, switch on the body’s antioxidant defenses, calm overactive immune signalling, restore the blood–milk barrier, and directly disrupt mastitis-causing bacteria. This narrative review integrates recent evidence on six major flavonoid groups, drawn predominantly from in vitro and murine experimental models, and explains how their shared and unique mechanisms relieve mammary inflammation, including emerging roles in gene regulation, ferroptosis and the gut–mammary axis. It offers researchers and veterinarians a clear framework for developing flavonoid-based strategies to protect the health of ruminants and humans.

## 1. Introduction

Mastitis is the most economically and clinically consequential disease of dairy production worldwide [1], and an important cause of morbidity in lactating women [2,3]. At the cellular level, it represents a stereotyped sequence of events: detection of pathogen-associated molecular patterns (PAMPs) by mammary epithelial cells and resident macrophages, activation of pro-inflammatory transcriptional programs, recruitment of neutrophils, generation of reactive oxygen species (ROS), and disruption of the blood–milk barrier [4,5,6]. When this response fails to resolve, parenchymal injury, fibrosis, and reduced lactational performance follow, with substantial consequences for animal welfare, milk quality, and on-farm antibiotic consumption.

Conventional management has relied heavily on antimicrobial chemotherapy, but the global trajectory toward antimicrobial stewardship has narrowed the scope of this approach [7,8,9]. Adjunct strategies that target the host’s inflammatory and redox response rather than the pathogen alone are therefore increasingly attractive. Within this space, flavonoids—a structurally heterogeneous family of plant-derived polyphenols—have accumulated substantial mechanistic and translational evidence supporting their role as modulators of inflammation, oxidative stress, and epithelial barrier function [10,11,12,13].

The therapeutic appeal of flavonoids in mastitis lies not in any single pharmacological action but in their capacity to engage multiple converging molecular nodes simultaneously. Across subclasses, individual compounds suppress canonical NF-κB and MAPK signaling, activate Nrf2-driven antioxidant defenses, restrain inflammasome assembly, preserve tight junctions, and, in several recently characterized cases, target bacterial virulence determinants or remodel the gut microbiota in ways that reverberate systemically to the mammary gland [13,14,15,16].

Existing reviews have largely enumerated individual compounds in isolation, often with considerable overlap and limited mechanistic integration across subclasses. In contrast, the present review adopts a deliberately synthetic approach: a unifying molecular framework is established first, followed by a focused treatment of each subclass that highlights only those mechanisms or contexts contributing genuinely novel insight. Three figures anchor the discussion by depicting the integrated signaling architecture, subclass-specific mechanistic emphasis, and translational implications of flavonoid action. Collectively, this organization is intended to provide a coherent, redox-centered synthesis of contemporary evidence relevant to investigators in mammary biology, veterinary medicine, and translational redox science.

## 2. Literature Search Strategy

To improve transparency and reproducibility, a structured search strategy was used to identify the literature synthesized in this review. PubMed, Scopus, Web of Science and Google Scholar were searched for records published between January 2014 and June 2026, using combinations of the terms “flavonoid”, “mastitis” and “mammary gland inflammation”, together with individual compound names (e.g., quercetin, myricetin, kaempferol, rutin, fisetin, luteolin, apigenin, baicalin, baicalein, wogonin, diosmetin, hesperetin, naringenin, daidzein, puerarin, isoliquiritigenin, EGCG) and mechanistic terms (e.g., NF-κB, MAPK, Nrf2, NLRP3, ferroptosis, gut microbiota, blood–milk barrier, antimicrobial resistance). Reference lists of retrieved articles and recent reviews were hand-searched for additional relevant studies. Original research articles reporting flavonoid effects on mastitis-relevant endpoints in cell-culture, rodent, or ruminant models were prioritized, and records were screened for relevance, English-language availability and methodological clarity. As this is a narrative rather than a systematic review, the search was not exhaustive and a formal risk-of-bias appraisal was not undertaken; the objective was instead to provide a representative, mechanistically organized synthesis of the most pertinent and recent literature in this rapidly evolving field.

## 3. Scope, Evidence Hierarchy, and a Note on Cross-Species Context

This review is centered on mastitis in dairy ruminants—principally cattle, sheep and goats—given the substantial differences in productivity demands, predominant pathogens and herd-level control strategies relative to human lactation. Mammary gland inflammation is also a recognized clinical entity in lactating women [2,3], and the discussion occasionally draws on translational or human-derived mechanistic findings that directly illuminate a pathway relevant to ruminant mastitis. This reflects the underlying evidence base rather than a claim of clinical equivalence between species: the large majority of the mechanistic findings synthesized here, across all six flavonoid subclasses, were generated in vitro, in bovine or murine mammary epithelial cell lines, or in murine mastitis models—experimental systems that are not species-specific and are widely used to establish redox and innate-immune signalling principles broadly conserved across mammals, including both ruminants and humans. Confirmatory evidence in dairy cattle, sheep or goats is considerably more limited than the in vitro and murine literature and is explicitly flagged throughout the text and summarized, alongside the principal experimental model for each compound. Cross-species mechanistic parallels are accordingly presented for context only: they do not constitute, and should not be interpreted as, direct evidence of clinical efficacy in either ruminants or humans, and dedicated ruminant-specific efficacy trials—together with separate, purpose-designed human clinical studies where relevant—will be required before such extrapolation can be drawn. The six flavonoid subclasses considered here are obtained predominantly from citrus fruits (flavanones), onions, kale, berries and tea (flavonols), parsley, celery and *Scutellaria* species (flavones), soybean and *Astragalus* species (isoflavones), *Glycyrrhiza* (licorice) species (chalcones) and green tea (flavan-3-ols); the principal dietary or botanical source is identified at first mention of each compound class in the sections below.

## 4. Molecular Pathogenesis of Mammary Gland Inflammation

**Figure 1:**
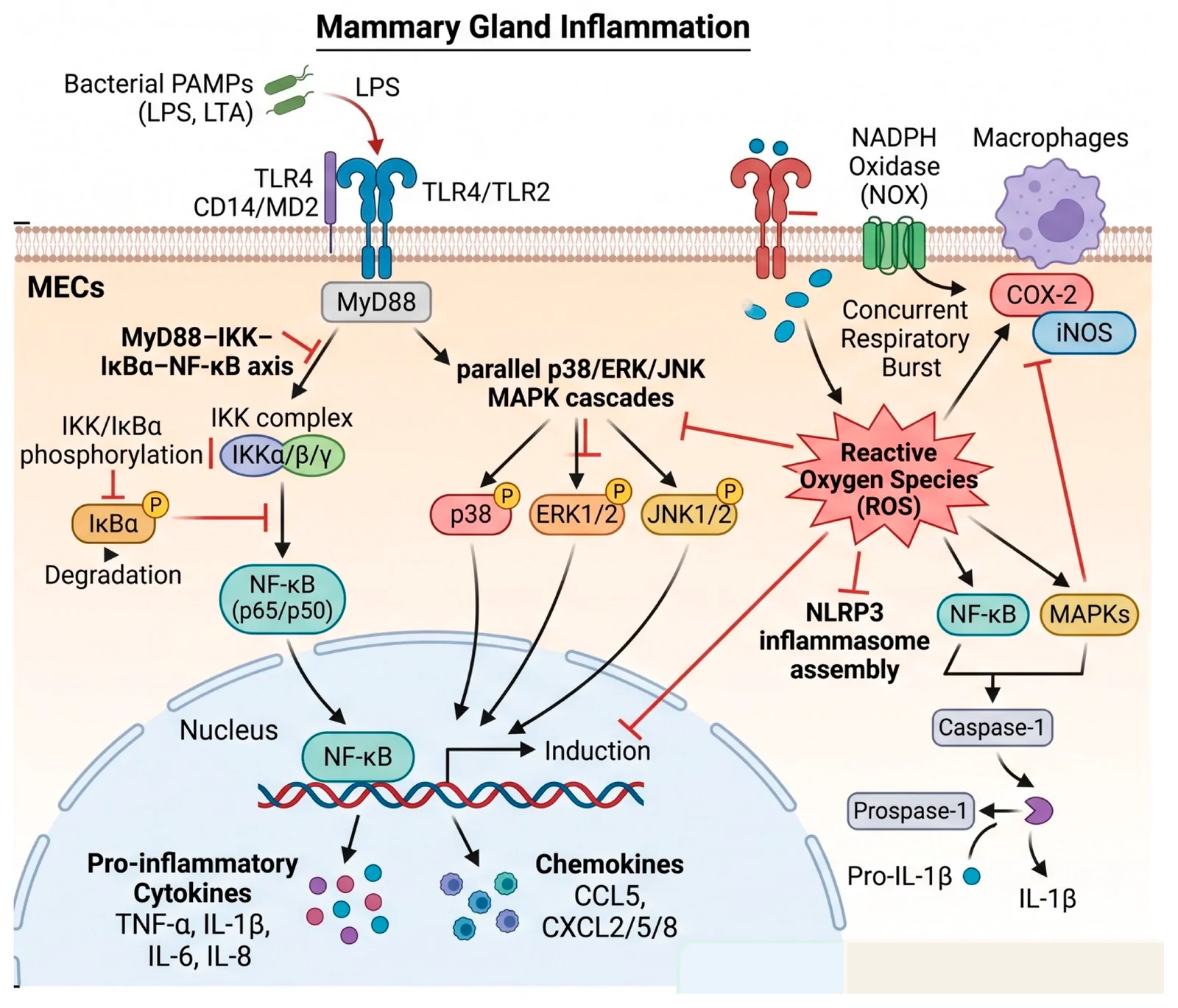
Pathogen recognition and inflammatory signaling underlying mammary gland inflammation. Bacterial PAMPs—lipopolysaccharide (LPS) and lipoteichoic acid (LTA)—are recognized by TLR4/CD14/MD2 and TLR2 on mammary epithelial cells. MyD88 recruitment activates two parallel arms: IKK-dependent degradation of IκBα with nuclear translocation of NF-κB (p65/p50), and phosphorylation of p38, ERK1/2 and JNK. NADPH oxidase-derived reactive oxygen species (ROS) potentiate both arms and promote NLRP3 inflammasome assembly and caspase-1-dependent maturation of IL-1β. The combined transcriptional output comprises pro-inflammatory cytokines and chemokines, culminating in an inflamed mammary gland.

Mastitis develops when mastitis-causing bacteria breach the teat canal and colonize the mammary parenchyma, where the balance between pathogen virulence and the host innate immune response determines the severity of disease [1,4,7]. The principal aetiological agents span environmental and contagious organisms, including *Escherichia coli*, *Staphylococcus aureus*, *Streptococcus agalactiae*, *Streptococcus uberis* and coagulase-negative staphylococci [1,7]. Although the inciting pathogens differ in their surface molecules and virulence strategies, they converge on a largely stereotyped molecular cascade, summarized in Figure 1 and detailed below.

The initiating molecular event is the recognition of pathogen-associated molecular patterns (PAMPs) by mammary epithelial cells and resident macrophages [4,5,6]. Lipopolysaccharide of Gram-negative bacteria is sensed by the TLR4/CD14/MD2 complex, whereas lipoteichoic acid of Gram-positive bacteria engages TLR2. Receptor engagement recruits the adaptor MyD88 and activates the IκB kinase (IKK) complex, driving phosphorylation-dependent degradation of IκBα and nuclear translocation of NF-κB (p65/p50); in parallel, the p38, ERK1/2, and JNK MAPK cascades activate AP-1 [17,18,19,20,21,22,23,24,25,26,27]. Together, these transcription factors orchestrate the production of pro-inflammatory cytokines (TNF-α, IL-1β, IL-6, IL-8) and chemokines (CCL5, CXCL2/5/8) that define the acute inflammatory phase.

Chemokines release recruits circulating neutrophils into the gland, and their NADPH-oxidase-driven respiratory burst generates reactive oxygen species (ROS) intended to kill invading bacteria [4,6]. Concurrently, assembly of the NLRP3 inflammasome activates caspase-1, which matures pro-IL-1β and pro-IL-18 and, when sustained, triggers gasdermin-D-dependent pyroptosis of epithelial and immune cells [27,28,29,30,31]. This amplification step converts a contained, resolving response into a self-perpetuating one when bacterial clearance is incomplete.

When ROS generation outstrips the enzymatic and non-enzymatic antioxidant defences of the gland, oxidative stress ensues [32,33,34,35,36,37]. Excess ROS are not merely cytotoxic by-products; they activate the redox-sensitive NF-κB and MAPK pathways, thereby amplifying cytokine production and establishing a feed-forward inflammatory–oxidative loop central to the transition from transient to chronic injury [38,39]. Lipid peroxidation and dysregulated iron handling can additionally drive ferroptotic death of mammary epithelial cells, an iron-dependent form of regulated cell death increasingly recognized as a contributor to tissue damage in bacterial mastitis [13].

A defining structural consequence of this cascade is disruption of the blood–milk barrier. Pro-inflammatory mediators and ROS degrade the tight junction proteins ZO-1, occludin, and claudin-3/4, increasing paracellular permeability, allowing serum components and somatic cells to enter the milk, and raising the somatic cell count [4,6]. Failure to resolve the response leads to parenchymal damage, fibrosis, apoptosis of secretory epithelium and a sustained fall in milk yield and quality, the clinical and economic hallmarks of mastitis.

Finally, susceptibility to this local cascade is shaped by systemic factors, most notably the metabolic and oxidative stress of the periparturient period and the composition of the gut microbiota, which conditions systemic immune tone and is causally linked to mammary inflammation through the gut–mammary axis [1]. Taken together, pathogen sensing, NF-κB/MAPK-driven cytokine production, neutrophilic oxidative burst, inflammasome activation, redox imbalance and blood–milk barrier failure constitute the interconnected molecular nodes of mammary gland inflammation, and it is precisely these nodes that the flavonoids discussed in the following sections are able to engage.

## 5. A Unifying Molecular Framework for Flavonoid Action in Mammary Inflammation

### 5.1. The Inflammatory Backbone: TLR4–NF-κB and MAPK Signaling

The TLR4/TLR2–MyD88–IKK–NF-κB axis and the parallel p38/ERK1/2/JNK–AP-1 cascades detailed in Section 4 (Figure 1) together constitute the inflammatory backbone on which flavonoids act [17,18,19,20,21]. Flavonoids interrupt this backbone at multiple points: by reducing TLR4/CD14/MD2/MyD88 expression, inhibiting IKK and IκBα phosphorylation, blocking p65 nuclear translocation, and attenuating MAPK phosphorylation [5,12,40]. The same TLR2/NF-κB axis is also targeted by structurally related homoisoflavonoids such as brazilin during *S. aureus*-induced mastitis [41]. 

Two qualifications temper this apparent uniformity. First, the level of the cascade at which inhibition occurs differs between compounds and is more often inferred than demonstrated. Receptor-proximal effects are reported for quercetin [15] and AKT- or IKK-proximal effects for myricetin, wogonin, licochalcone A and farrerol [42,43,44,45], and one direct physical interaction—naringenin with IκBα—rests on molecular docking alone [46]. Only a minority of studies apply pharmacological or genetic loss-of-function controls (Compound C for AMPK [47], BAY 11-7028 for NF-κB [5], CH223191 for AhR [48], EX-527 for SIRT1 [48,49], siRNA for Nrf2 [50], IL-17RA-knockout mice [51]; elsewhere, pathway attribution rests on correlative changes in phosphoprotein abundance and should be treated as provisional. Second, NF-κB and MAPK signaling are also required for neutrophil recruitment and bacterial clearance, so suppression of this backbone is not unconditionally beneficial. Nearly all studies reviewed here quantify inflammatory mediators rather than bacterial burden, and the possibility that potent NF-κB inhibition delays elimination of intramammary pathogens has not been formally excluded (Section 12.7).

### 5.2. Effector and Barrier-Level Mechanisms

Downstream of transcriptional regulation, cyclooxygenase-2 (COX-2) and inducible nitric oxide synthase (iNOS) generate prostaglandins and large quantities of nitric oxide that drive vasodilation, pain, and tissue damage [52]. Flavonoids suppress both their expression and, for several compounds, their enzymatic activity [16,53,54]. The NLRP3 inflammasome—which cleaves pro-IL-1β and pro-IL-18 through caspase-1—represents an additional therapeutic node, with several flavonoids (notably baicalein and naringenin) restraining its assembly [27,28,29,30,31]. Finally, restoration of tight-junction proteins (ZO-1, occludin, claudin-3/4) repairs the blood–milk barrier, which increasingly serves as a functional readout of flavonoid efficacy in vivo [30].

### 5.3. The Redox Axis: ROS Generation, Nrf2/Keap1/ARE, and HO-1

Oxidative stress occupies a central, self-perpetuating position in mammary inflammation: the phagocyte respiratory burst described in Section 4 generates ROS in quantities that overwhelm local enzymatic and non-enzymatic antioxidant defenses, damaging mammary epithelial cells, compromising tight-junction integrity and the blood–milk barrier, and reducing milk yield and quality [4,6,32,33,34,35,36,37,38]. Redox and inflammatory signaling are mutually reinforcing: ROS activate NF-κB and MAPK; the resulting mediators (TNF-α, IL-1β, IL-6, COX-2, iNOS) recruit further phagocytes; and the resulting feed-forward loop converts transient inflammation into chronic injury, fibrosis, and apoptosis of secretory epithelium [39].

**Figure 2:**
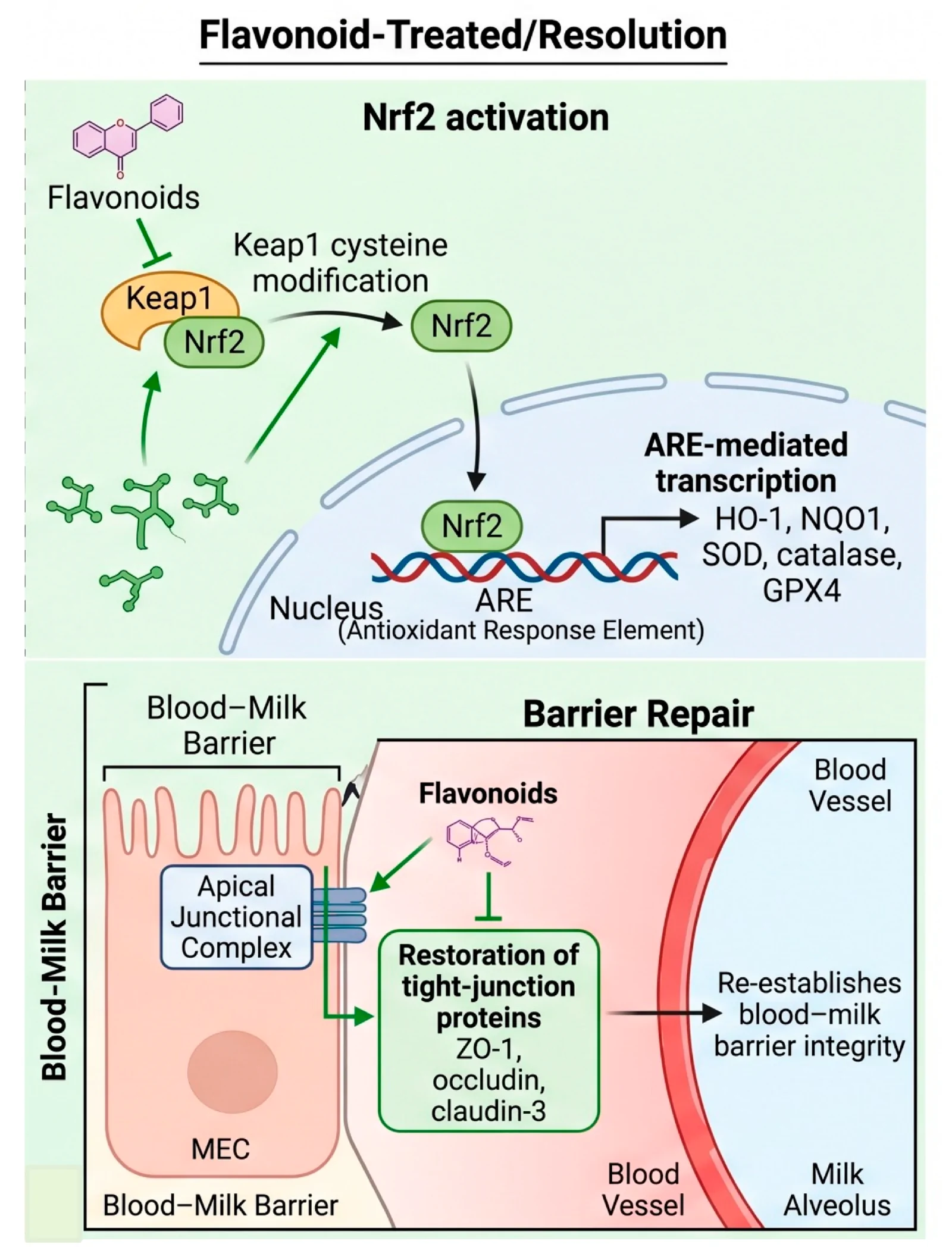
Flavonoid-mediated resolution of mammary gland inflammation: Nrf2 activation and blood–milk barrier repair. (Upper panel) Flavonoids interact with reactive cysteine residues on Keap1, releasing Nrf2, which enters the nucleus, binds the antioxidant response element (ARE) and drives transcription of cytoprotective genes (HO-1, NQO1, SOD, GPX4), thereby quenching ROS and dampening upstream inflammatory signaling. (Lower panel) Inflammation disrupts the apical junctional complex of mammary epithelial cells; flavonoids restore ZO-1, occludin and claudin-3, re-establishing blood–milk barrier integrity.

Flavonoids disrupt this pathological axis through complementary mechanisms [55]. Their polyphenolic structure—particularly the B-ring hydroxyl groups and catechol moiety—enables direct radical scavenging via hydrogen-atom transfer and single-electron donation, thereby neutralizing ROS before they propagate damage. Flavonoids also activate the Nrf2 pathway [56,57]. Under basal conditions, Nrf2 is sequestered by Keap1 and targeted for proteasomal degradation [58]. Flavonoids modify or interact with critical Keap1 cysteine residues, releasing Nrf2 to translocate to the nucleus, bind antioxidant response elements, and upregulate phase II detoxifying and antioxidant enzymes, including HO-1, NQO1, SOD, catalase, and glutathione peroxidase [58] (Figure 2).

By lowering intracellular ROS levels, flavonoids indirectly attenuate NF-κB and MAPK activation, thereby reducing downstream cytokine production. The HO-1 axis is particularly noteworthy: its byproducts—carbon monoxide, biliverdin, and ferritin-sequestered iron—exert independent anti-inflammatory and cytoprotective effects. Thus, flavonoids disrupt the underlying redox driver rather than merely suppressing symptoms, restoring mammary homeostasis. This dual capacity—direct radical-scavenging coupled with sustained transcriptional reprogramming of antioxidant defenses—positions flavonoids as promising natural agents for the prevention and adjunctive management of mammary gland inflammation in ruminant veterinary practice.

This positioning requires qualification on two counts. Direct radical scavenging measured in cell-free or cell-culture systems generally requires micromolar-to-millimolar concentrations that exceed the plasma concentrations achievable after oral dosing (Section 12.5), so the antioxidant effect observed in vivo is more plausibly dominated by Nrf2-mediated transcriptional reprogramming than by stoichiometric ROS quenching. In addition, the catechol and pyrogallol B-ring motifs responsible for electron donation also permit autoxidation and redox cycling in the presence of transition metals, such that several flavonoids behave as pro-oxidants at high concentrations—a possibility that has not been examined systematically in mammary tissue and that argues against assuming a monotonic dose–benefit relationship. Of the compounds reviewed, Nrf2 dependence has been confirmed by inhibitor or knockdown approaches only for baicalin [59], EGCG [50] and, indirectly, myricetin [60]; elsewhere it is inferred from expression changes alone.

### 5.4. Emerging Dimensions: Epitranscriptomics, Ferroptosis, and the Gut–Mammary Axis

Three dimensions warrant emphasis because they reframe the scope of flavonoid action. First, m^6^A-dependent post-transcriptional regulation, exemplified by quercetin’s modulation of CCL5 through YTHDF2 [61], introduces an epitranscriptomic layer of control. Second, ferroptosis—iron-dependent regulated cell death—is increasingly recognized as a contributor to bacterial mastitis, and several flavonoids (notably fisetin and puerarin) restrain it through Nrf2/GPX4/SLC7A11 and SIRT1/p53 signaling [13,49]. Third, gut microbiota composition affects systemic immune tone and, by extension, mammary inflammation; flavonoid-induced microbiota remodeling, causally validated by fecal microbiota transplantation (FMT), is a recurring theme particularly within the flavanone subclass [46,62,63]. The principal pathways modulated by flavonoids across subclasses are summarized in Table 1.

## 6. Flavonols

**Figure 3:**
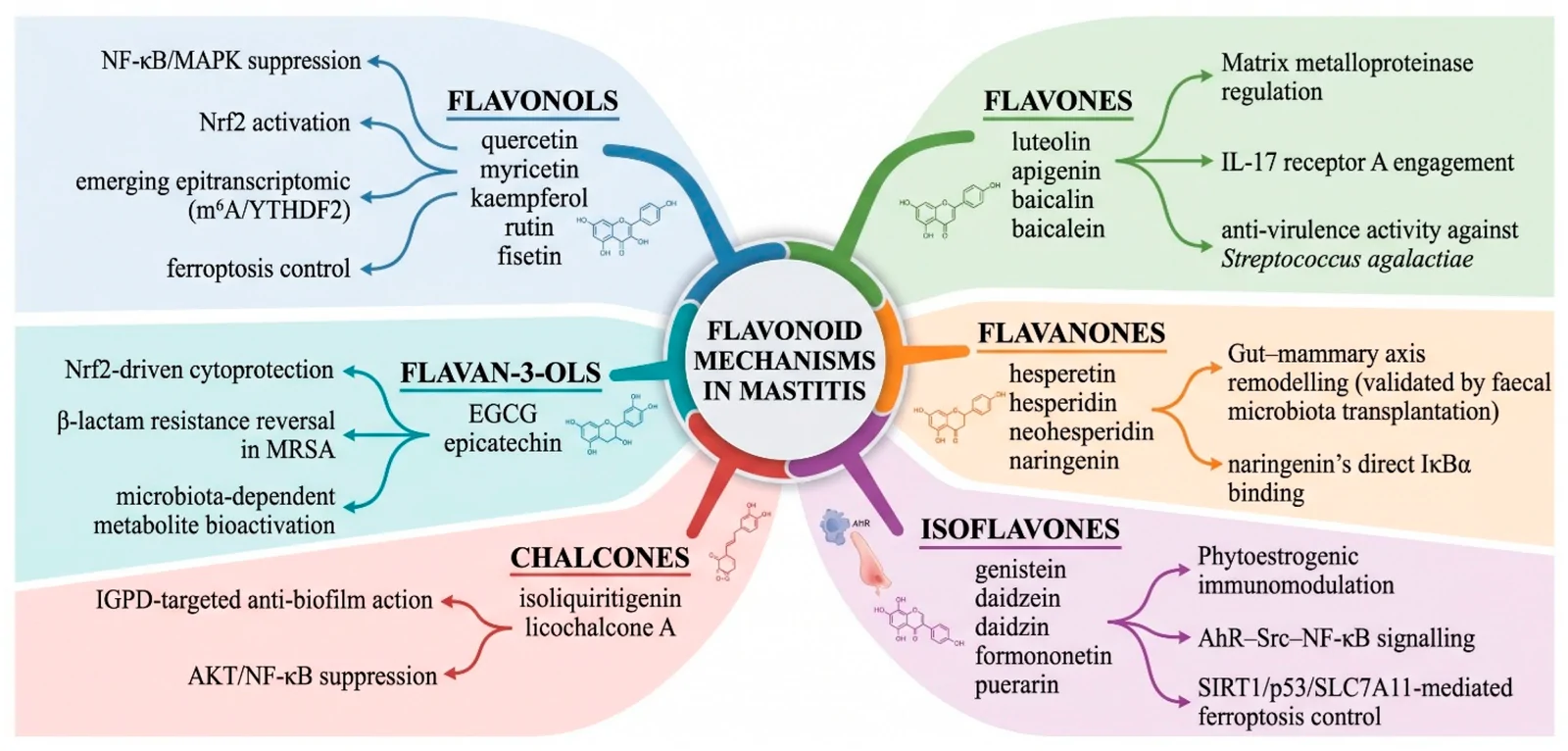
Subclass-specific emphasis of flavonoid action in mastitis. Six dietary flavonoid subclasses, each represented by its two best-evidenced compounds, are mapped against the three pathways engaged by all subclasses (NF-κB, MAPK, Nrf2/HO-1) and against the mechanism that distinguishes each subclass: epitranscriptomic and ferroptosis control (flavonols), IL-17RA engagement and anti-virulence activity (flavones), FMT-validated gut–mammary remodelling (flavanones), AhR–Src signalling and SIRT1-dependent ferroptosis control (isoflavones), IGPD-directed anti-biofilm activity (chalcones) and reversal of β-lactam resistance (flavan-3-ols).

Flavonols share a 3-hydroxyflavone backbone and are abundant in vegetables, fruits, berries, and tea. Within mammary inflammation, they collectively engage TLR4/NF-κB and MAPK signaling, Nrf2 activation, autophagy regulation, epitranscriptomic control, and direct anti-microbial action. The following discussion is restricted to mechanisms that distinguishes each compound; shared pathways are addressed only briefly to avoid redundancy with Section 2. An overview of the dominant mechanistic emphasis of each subclass is provided in Figure 3.

Quercetin (3,3′,4′,5,7-pentahydroxyflavone) is the most extensively studied flavonol in mastitis. In LPS-challenged bovine mammary epithelial cells (BMECs), it dose-dependently preserves viability and suppresses ROS, MDA, and a panel of cytokines and chemokines (TNF-α, IL-1β, IL-6, CXCL2/5/8, CCL5) by downregulating the TLR4/CD14/MD2/MyD88 complex and preventing NF-κB p65 nuclear translocation [15]. Network pharmacology converges on the PI3K-AKT and NF-κB axes as the dominant nodes [79]. Two further mechanisms distinguish quercetin from other flavonols. First, against a Gram-positive LTA challenge, quercetin restores tight-junction integrity in MAC-T cells and murine mammary tissue by suppressing dysregulated autophagy via AMPK/mTOR signaling, an effect reversed by the AMPK inhibitor Compound C [47]. Second, in *S. aureus*-induced bovine mastitis, quercetin operates at an epitranscriptomic level: YTHDF2-mediated m^6^A methylation of CCL5 mRNA reduces chemokine output and neutrophil recruitment, with concurrent effects on MMP1/3, IGFBP3, and biofilm formation [61]. Complementing these host-directed actions, quercetin-3-O-rhamnoside displays potent antibacterial (MIC 0.07–1.04 mg/mL), anti-quorum-sensing and anti-biofilm activity against mastitis-associated *S. aureus* [73].

Myricetin (3,3′,4′,5,5′,7-hexahydroxyflavone) is a broadly bioactive flavonol whose wider pharmacological profile spans anti-inflammatory, antioxidant, anticancer, and antimicrobial activities [80]. In the mammary context, myricetin operates through three partially independent axes. In LPS-induced mouse mammary tissue and mMECs, it dampens neutrophil infiltration, MPO activity, and cytokine output, and restores tight-junction proteins, primarily by inhibiting the AKT/IKK/NF-κB cascade [42]. This AKT-proximal site of action distinguishes it from quercetin’s receptor-proximal effect and may be particularly relevant when PI3K/AKT is constitutively activated. A parallel study from the same group identified suppression of p38 and ERK1/2 phosphorylation as an additional anti-inflammatory mechanism [81]. Critically, in H_2_O_2_-challenged BMECs, myricetin engages AMPK/Nrf2 signaling to enhance total antioxidant capacity and SOD activity, reduce ROS and MDA, and inhibit apoptosis [60]—a property of direct relevance to the metabolically oxidative periparturient window during which mastitis susceptibility peaks. Beyond host-directed activity, myricetin also exerts direct anti-virulence effects on key mastitis-relevant pathogens: against *Pseudomonas aeruginosa*, it inhibits biofilm formation, quorum sensing, and the production of pyocyanin and other virulence factors at sub-inhibitory concentrations [78], paralleling its activity against staphylococcal pathogens and supporting its dual host- and pathogen-directed therapeutic profile.

Kaempferol (3,4′,5,7-tetrahydroxyflavone) is mechanistically more compact: in LPS-induced murine mastitis, intraperitoneal kaempferol dose-dependently reduces neutrophil infiltration, MPO activity, and cytokine expression, with mechanistic effects converging on inhibition of NF-κB p65 phosphorylation and prevention of IκBα degradation [65]. A notable extension comes from the identification of angiopoietin-like protein 2 (ANGPTL2)—a mediator of vascular inflammation and tissue remodeling—as a kaempferol target in primary mammary epithelial cells [82], broadening kaempferol’s anti-inflammatory profile beyond classical cytokine suppression. Kaempferol additionally exhibits direct anti-biofilm activity against *Staphylococcus aureus*, inhibiting the primary attachment phase of biofilm formation and disrupting the early adhesion steps that precede the establishment of mature biofilms [77], positioning it, like quercetin, as a dual host- and pathogen-directed candidate.

Rutin (quercetin-3-O-rutinoside) extends quercetin’s profile with three context-specific actions. In LPS-induced murine mastitis, it suppresses NF-κB (reduced p-IKKβ/IKKβ, p-p65/p65, and nuclear p65) and modulates the ER stress chaperone GRP78, indicating crosstalk between inflammatory and ER stress signaling [83]. In LPS-treated BMECs, rutin activates SIRT1, which in turn restrains NLRP3 inflammasome assembly and gasdermin D-mediated pyroptosis; EX-527-mediated inhibition of SIRT1 abolishes these protective effects [71]. In transition Hu sheep, dietary rutin reduces serum BHBA, H_2_O_2_, and MDA while elevating CAT, GSH-Px, SOD, and T-AOC, with Nrf2/HO-1 upregulation and suppression of pro-apoptotic markers demonstrating periparturient cytoprotection [33,69]. Finally, rutin (0.8 mg/mL) inhibits *Staphylococcus xylosus* biofilm formation by directly binding imidazole glycerol phosphate dehydratase (IGPD), the rate-limiting enzyme of histidine biosynthesis [75]—a target it shares with isoliquiritigenin (Section 7).

Fisetin (3,3′,4′,7-tetrahydroxyflavone) is a comparatively under-studied but mechanistically distinctive flavonol. In LPS-induced MAC-T cell and murine mastitis models, it suppresses IL-1β, TNF-α, and IL-6 and simultaneously engages the Nrf2/Keap1/HO-1 antioxidant axis and the GPX4/xCT ferroptosis pathway, thereby lowering tissue iron and MDA levels while raising SOD and total antioxidant capacity [13]. Beyond the mammary epithelium, fisetin reshapes the gut microbiota composition and short-chain fatty acid output, with these systemic changes correlating with the restoration of blood–milk barrier integrity. 

## 7. Flavones

Flavones (2-phenylchromen-4-one backbone, no C3-hydroxyl) share substantial mechanistic ground with flavonols but display several distinguishing features, including matrix-metalloproteinase regulation, IL-17 receptor A engagement, anti-virulence activity against *Streptococcus agalactiae,* and, in selected compounds, ferroptosis inhibition. Their general antibacterial properties are reviewed elsewhere [84].

Luteolin (3′,4′,5,7-tetrahydroxyflavone) is among the most abundant dietary flavones. In *Staphylococcus aureus*-induced murine mastitis, it reduces histopathological injury, neutrophil infiltration, and cytokine output, while downregulating TLR2/TLR4 and inhibiting IκBα and p65 phosphorylation [64]. A distinctive feature is its concurrent suppression of MMP-2 and MMP-9 and induction of TIMP-1 and TIMP-2, preserving extracellular matrix integrity and contributing independently to blood–milk barrier maintenance. Subsequent BMEC work extended this mechanism to include attenuation of p38, ERK, and JNK phosphorylation [66], establishing dual NF-κB/MAPK blockade across species and model systems.

Apigenin (4′,5,7-trihydroxyflavone) is the principal bioactive of *Taraxacum officinale* and *Barleria cristata*. In an intra-nipple LPS instillation model in postpartum Sprague–Dawley rats—methodologically closer to clinical bovine intramammary infection than most murine models—apigenin attenuates histopathological lesions, MPO activity, and TNF-α/IL-1β/IL-6 expression, with the mechanism rigorously delineated through co-treatment with the NF-κB inhibitor BAY 11-7028, demonstrating predominant action via the TLR4/NF-κB axis [5].

Baicalin and baicalein—glucuronide and aglycone, respectively, both principal flavones of *Scutellaria baicalensis*—illustrate how glycosylation diversifies pharmacological emphasis within a closely related pair. Baicalin engages an IL-17 receptor A (IL-17RA)-centric mechanism: in a particularly rigorous dual-species design—lactating mice challenged via the intramammary route with 10^7^ CFU *E. coli* and Holstein cows challenged with 6 × 10^6^ CFU/mL—oral or systemic baicalin reduced tissue damage, suppressed pro-inflammatory cytokines and preserved the blood–milk barrier; network pharmacology and multi-omics identified IL-17RA as the primary target, validated by phenocopying in IL-17RA knockout mice [85]. Baicalin also engages Nrf2-dependent cytoprotection in H_2_O_2_-challenged BMECs, with retinoic acid-mediated Nrf2 inhibition reversing its protective effects [59]. In ovine mammary epithelial cells, baicalin (25 µg/mL) enhances proliferation, antioxidant capacity, and milk fat/lactose synthesis genes [51], suggesting a dual productivity-and-health benefit in small ruminant dairy systems. Baicalein, by contrast, operates predominantly through anti-virulence rather than direct bactericidal action. At sub-inhibitory concentrations (≥4 µg/mL), despite MICs against *Streptococcus agalactiae* exceeding 1024 µg/mL, baicalein reduces GBS invasion of BMECs by >50%, downregulates the surface adhesin pbsP, suppresses nucleotide biosynthesis and energy metabolism (raising the NADH/NAD^+^ ratio and lowering ATP), and concurrently activates host Nrf2 and reduces NF-κB/IL-6 [76]. This integrated host–pathogen mechanism is highly distinctive within the flavonoid landscape.

Wogonin (5,7-dihydroxy-8-methoxyflavone), another major flavone of *Scutellaria baicalensis*, complements the baicalin/baicalein pair through a coordinated anti-inflammatory and antioxidant profile. In LPS-induced murine mastitis and primary mammary epithelial cells, wogonin attenuates histopathological injury, neutrophil infiltration, and pro-inflammatory cytokine output by inhibiting the Akt/NF-κB pathway while simultaneously activating Nrf2/HO-1 signaling and bolstering endogenous antioxidant defenses [44]. This dual engagement of an Akt-proximal inflammatory node and the Keap1/Nrf2 redox node mirrors the integrated mechanism seen with myricetin and neohesperidin, reinforcing that simultaneous inflammatory–oxidative interruption is a recurrent property of high-potency flavone aglycones.

Diosmetin (5,7,3′-trihydroxy-4′-methoxyflavone), a methylated derivative of luteolin abundant in citrus peel, occupies a mechanistically distinctive niche centered on ferroptosis control. In *Staphylococcus aureus*-induced murine mastitis and BMECs, diosmetin alleviates inflammatory injury and restores mammary tissue architecture by inhibiting SIRT1/GPX4-mediated ferroptosis, with concomitant reductions in lipid peroxidation, iron accumulation and MDA, and restoration of glutathione and GPX4 activity [72]. The SIRT1/GPX4 axis identified for diosmetin closely parallels the SIRT1/p53/SLC7A11 axis described for puerarin (Section 6) and the Nrf2/GPX4/xCT route for fisetin (Section 3), collectively establishing ferroptosis suppression as an emerging, cross-subclass mechanism of flavonoid action in bacterial mastitis.

## 8. Flavanones

Flavanones (saturated C2–C3 bond, chiral C2) are abundant in citrus and recurrently implicate the gut–mammary axis as a contributory mechanism. Across hesperetin, neohesperidin, and naringenin, FMT experiments causally validate microbiota remodeling as an independent protective mechanism rather than a correlate of intramammary action—a methodological standard rarely achieved for other subclasses.

Hesperetin and hesperidin are interconvertible through intestinal deglycosylation (hesperidin = hesperetin-7-O-rutinoside). In LPS-induced murine mastitis, oral hesperetin alleviates histopathology, suppresses cytokine production, and preserves blood–milk barrier integrity through TLR4/NF-κB inhibition and restoration of tight-junction proteins [62]. Critically, FMT from hesperetin-treated donors ameliorates mastitis in naïve recipients, providing causal evidence for microbiota-dependent protection. Recent comparative work has further delineated the concentration-dependent bioactivity profile of hesperetin: at low concentrations, it acts predominantly as an antioxidant and anti-inflammatory agent, whereas at higher concentrations it exhibits direct antibacterial activity, establishing dose as a key determinant of the dominant pharmacological action [86]. Translationally, in a pilot clinical study of twelve Polish Holstein–Friesian cows with active quarter-level mastitis, intramammary hesperidin (30 mg/quarter/day)—alongside parallel arms of chrysin and naringenin—significantly reduced milk somatic cell count without adverse hematological or biochemical effects [87], constituting one of the few direct clinical demonstrations of flavanone efficacy in lactating dairy cattle.

Neohesperidin, the 7-O-neohesperidoside of hesperetin, engages a particularly comprehensive multi-target profile: simultaneous TLR4/NF-κB suppression and AMPK/Nrf2/HO-1 induction in mammary epithelial cells, coupled with FMT-validated gut microbiota remodeling and blood–milk barrier preservation [63]. The concurrent engagement of pro-inflammatory and antioxidant pathways mirrors that of rutin and may be a generalisable property of C7-glycosylated flavanones.

Naringenin and naringin together illustrate the aglycone–glycoside continuum within the flavanone subclass. Naringenin (4′,5,7-trihydroxyflavanone) is distinguished mechanistically by a direct, high-affinity physical interaction with IκBα: molecular docking analyses identify a binding energy of −24.05 kcal/mol, with naringenin sterically blocking IκBα phosphorylation and thus the initiating step of canonical NF-κB activation [46]. In bovine and murine MECs, this produces dose-dependent suppression of TNF-α, IL-1β, IL-6, and IL-8. In vivo, naringenin pretreatment (3.5 µg/g once daily for seven days prior to intramammary LPS challenge) reduces mammary swelling, neutrophil infiltration, and acinar damage in mice, with 16S rRNA sequencing showing reversal of LPS-induced gut dysbiosis (restored Firmicutes/Bacteroidetes ratio) and corresponding restoration of tight junctions. Concentration-dependent profiling further indicates that naringenin, like hesperetin, shifts from primarily antioxidant/anti-inflammatory effects at low concentrations to direct antibacterial effects at higher concentrations [86]. Its 7-O-neohesperidoside, naringin, extends these effects systemically: dietary supplementation in transition dairy cows improves systemic metabolic status and alleviates oxidative stress through modulation of adipose tissue function, lowering NEFA and inflammatory markers, and improving the redox balance during the periparturient window [88], a context in which mastitis susceptibility is greatest. Together, the direct binding evidence, the gut-microbiota effect, and the periparturient metabolic benefit establish the naringenin–naringin pair as one of the most mechanistically precise flavanone candidates for mastitis prevention.

Farrerol (2,3-dihydro-5,7-dihydroxy-6,8-dimethyl-2-(4-hydroxyphenyl)-4H-chromen-4-one), a 6,8-dimethylated dihydroflavone characteristic of *Rhododendron* species, provides a further example of an aglycone flavanone with mammary-protective activity. In LPS-induced murine mastitis, farrerol attenuates histopathological injury, MPO activity, and pro-inflammatory cytokine expression by simultaneously inhibiting the Akt/NF-κB p65, Erk1/2, and p38 signaling axes [45]. This combined Akt and dual-MAPK suppression places farrerol mechanistically alongside myricetin and licochalcone A as flavonoids that engage both inflammatory and survival kinase nodes, and reinforces dihydroflavones as a structurally distinct but pharmacologically convergent flavanone variant.

## 9. Isoflavones

Isoflavones, in which the B-ring is attached at C3 rather than C2, are structurally related to 17β-estradiol and are most abundant in soybean and Astragalus species. Their mastitis-relevant repertoire encompasses host-directed anti-inflammatory and antioxidant activity, pathogen-directed interference with virulence, AhR-mediated signaling, and SIRT1-dependent control of ferroptosis.

Soybean isoflavones, daidzein, and daidzin together demonstrate both mixture-level and compound-level efficacy. Mixed soybean isoflavone (SI) treatment of BMECs (60–80 µg/mL) enhances viability and inhibits *Streptococcus agalactiae* growth and internalization, with proteomics identifying 222 differentially expressed proteins [89]. Upregulated p27kip1, PIK3CA, and CD82 collectively reflect enhanced stress-adaptive, survival, and anti-inflammatory tone, while downregulated integrin β5 and osteopontin reduce bacterial adhesion. KEGG enrichment in Fc-gamma receptor-mediated phagocytosis and complement cascade pathways indicates a primed innate immune state. Within this mixture, daidzein and its glucoside, daidzin, exhibit a clear aglycone–glycoside divergence. Both suppress NO, IL-6, TNF-α, COX-2, and iNOS in LPS-stimulated RAW264.7 macrophages and inhibit p38/ERK phosphorylation [67], but only daidzein blocks the complete IKKα/β–IκBα–p65 phosphorylation sequence and p65 nuclear translocation—producing effects comparable to dexamethasone. This profile, consistent with the superior membrane permeability of the aglycone, has formulation implications: intestinal deglycosylation of daidzin to daidzein may be a prerequisite for full activity. Mammary-specific work confirms that daidzein suppresses MAPK/NF-κB signaling and reduces IL-6 and IL-1β in LPS-stimulated mouse mammary epithelial cells [22]. In a heat-stress context, dietary daidzein (300–400 mg/day for 60 days) in late-lactation dairy cows elevates serum IgG, IFN-α, and IL-2 [90], restoring immunocompetence in a setting of recognized mastitis predisposition.

Genistein and equol—the parent isoflavone and its microbial metabolite—attenuate IL-12/IL-18-induced IFN-γ production by NK cells through MAPK pathway suppression and reduced IL-18Rα upregulation [91]. Although NK-cell suppression seems counterintuitive in the context of infection, restraint of immunopathological amplification is relevant to subclinical and chronic mastitis. Equol bioactivation depends on the composition of the individual gut microbiota, introducing inter-individual variability that complicates translation.

Formononetin (7-hydroxy-4′-methoxyisoflavone), the principal isoflavone of *Radix Astragali*, is alone among the flavonoids reviewed here in operating through the aryl hydrocarbon receptor (AhR). In LPS-induced murine mastitis and EpH4-Ev cells, formononetin attenuates histopathology, MPO activity, and pro-inflammatory cytokine production; upregulates tight junction proteins; and inhibits NF-κB signaling [48]. Mechanistic dissection shows that AhR activation inactivates Src kinase, an upstream activator of NF-κB; the selective AhR antagonist CH223191 reverses both effects, providing chemical-genetic target validation. The AhR–Src–NF-κB axis is mechanistically distinct from all other flavonoids reviewed.

Puerarin (daidzein-8-C-glucoside) bears a C8-linked glucose, an unusual feature among isoflavones, conferring resistance to intestinal deglycosylation. In H_2_O_2_-challenged BMECs, it elevates GSH, SOD, CAT and total antioxidant capacity through Nrf2/HO-1/xCT upregulation; suppresses NF-κB-mediated mediators (IL-6, IL-8, CCL5); and restores claudin-4, occludin and ZO-1 [70]. Translationally, mastitic dairy cows supplemented with puerarin show significantly reduced milk and serum TNF-α, IL-6 and IL-1β. In *S. aureus*-induced murine mastitis, puerarin additionally inhibits ferroptosis: it upregulates SIRT1 and SLC7A11 and downregulates p53, thereby restoring glutathione and GPX4 activity [49]; EX-527-mediated inhibition of SIRT1 abolishes the protective effect. The linear mechanistic sequence—puerarin → SIRT1 → ↓p53 → ↑SLC7A11 → restored GSH/GPX4 → ferroptosis suppression—is among the most clearly delineated cell-death-targeting mechanisms described for any flavonoid in mastitis, and parallels the SIRT1/NLRP3 axis of rutin (Section 3) and the SIRT1/GPX4 axis of diosmetin (Section 4).

## 10. Chalcones

Chalcones, biosynthetically the open-chain precursors to all closed-ring flavonoids, possess an α, β-unsaturated carbonyl system that confers electrophilic reactivity and broad protein-binding capacity. Two compounds dominate the mastitis literature.

Isoliquiritigenin (ISL) (4,2′,4′-trihydroxychalcone), the principal chalcone of *Glycyrrhiza uralensis*, is a bifunctional agent. Against *Staphylococcus xylosus* it has an MIC of 80 µg/mL, with sub-MIC concentrations significantly inhibiting biofilm formation; bio-layer interferometry confirms direct ISL–IGPD binding (K_D = 234 µM), downstream reductions in hisB mRNA and IGPD protein, and suppression of intracellular histidine and biofilm matrix integrity [74]. In vivo, ISL reduces TNF-α and IL-6 and reverses mammary histopathological damage. The fact that ISL and the structurally unrelated flavonol rutin both converge on IGPD is mechanistically striking: IGPD emerges as a druggable, generalizable vulnerability across coagulase-negative staphylococcal mastitis pathogens. In LPS-challenged MAC-T cells, ISL (2.5–10 µg/mL) additionally suppresses COX-2, iNOS, and pro-inflammatory cytokine expression, with a mechanism characterized by reduced phosphorylation of p65, IκBα, p38, ERK, and JNK [54]. The breadth of ISL’s antioxidant and anti-inflammatory action extends beyond the mammary gland: in LPS-induced cognitive impairment models, ISL similarly suppresses systemic oxidative stress and inflammatory cytokine release, supporting its capacity to act on the broader LPS–NF-κB–ROS axis [92], which reinforces the translational case for chalcone-based redox modulation in periparturient mastitis.

Licochalcone A, a retrochalcone of *G. uralensis* with additional methoxylation and prenylation, exerts AKT/NF-κB and MAPK blockade in LPS-induced mouse mastitis and corresponding mMECs, while upregulating ZO-1, occludin, and the mammary-enriched claudin-3 [43]. The combined AKT- and MAPK-proximal action distinguishes licochalcone A from ISL and parallels the myricetin AKT/IKK/NF-κB profile, illustrating how structurally distinct flavonoids converge on the same upstream signalling node.

## 11. Flavan-3-ols (Catechins)

Flavan-3-ols are characterized by C3 hydroxylation, lack of a C4 carbonyl and strong hydrogen-bonding and metal-chelating capacity. Green tea provides the principal dietary catechins, with oligomeric proanthocyanidins representing their polymerized counterparts in fruits, seeds, and bark.

Epigallocatechin-3-gallate (EGCG), the most studied catechin in mammary contexts, operates through Nrf2-dependent cytoprotection, NF-κB/MAPK suppression, and a notable resistance-reversal mechanism against MRSA. In H_2_O_2_-challenged BMECs, EGCG (5 µM) drives Nrf2 nuclear translocation—siRNA-mediated Nrf2 knockdown reverses its protective effect—and concurrently inhibits p38 MAPK and downstream NF-κB/caspase-3 activation [50]. In LPS-challenged bovine hepatocytes and BALB/c mice, EGCG (50 µM) inhibits NF-κB (p65, IκBα) and MAPK (p38, ERK, JNK) phosphorylation, reduces p65 binding to inflammatory gene promoters and restores antioxidant enzyme activity [68]. In LPS-induced rat mastitis, EGCG additionally suppresses HIF-1α overexpression [93]— a transcription factor relevant to the hypoxic mammary microenvironment of acute infection. A mechanistically distinctive property of galloylated catechins is their capacity to phenotypically reverse MRSA β-lactam resistance, lowering MICs from 256–512 to ~1 mg/L through cytoplasmic membrane intercalation that disperses peptidoglycan synthesis proteins, suppresses biofilm formation, and disrupts virulence protein secretion [94]. Given the increasing prevalence of MRSA in bovine mastitis, this resistance-reversing rather than directly bactericidal mode of action carries clear translational implications for combination therapy. Comparative work across EGCG, EGC, ECG and EC indicates that C3 gallate esterification enhances anti-inflammatory potency and that catechins are preferentially active against Gram-positive over Gram-negative mastitis pathogens [95]. Head-to-head comparison with hydroxytyrosol in BME-UV1 cells suggests complementary profiles—stronger antioxidant action for hydroxytyrosol, predominantly anti-inflammatory action for EGCG—supporting combinatorial formulation strategies [96]. 

Epicatechin, the simplest flavan-3-ol, suppresses NF-κB, MAPK, and JAK/STAT signaling and indirectly activates Nrf2, with a profile broadly consistent with EGCG but generally lower potency [97]. A conceptually important distinction is that colonic microbial metabolites of epicatechin display enhanced bioactivity relative to the parent compound, and inter-individual variation in gut microbiota composition generates distinct metabotypes that may underlie variable clinical responses. This microbiota-dependent bioactivation parallels the gut–mammary mechanisms described in Section 5 and reinforces the broader principle that in vivo flavonoid efficacy is shaped substantially by the microbiome.

**Figure 4:**
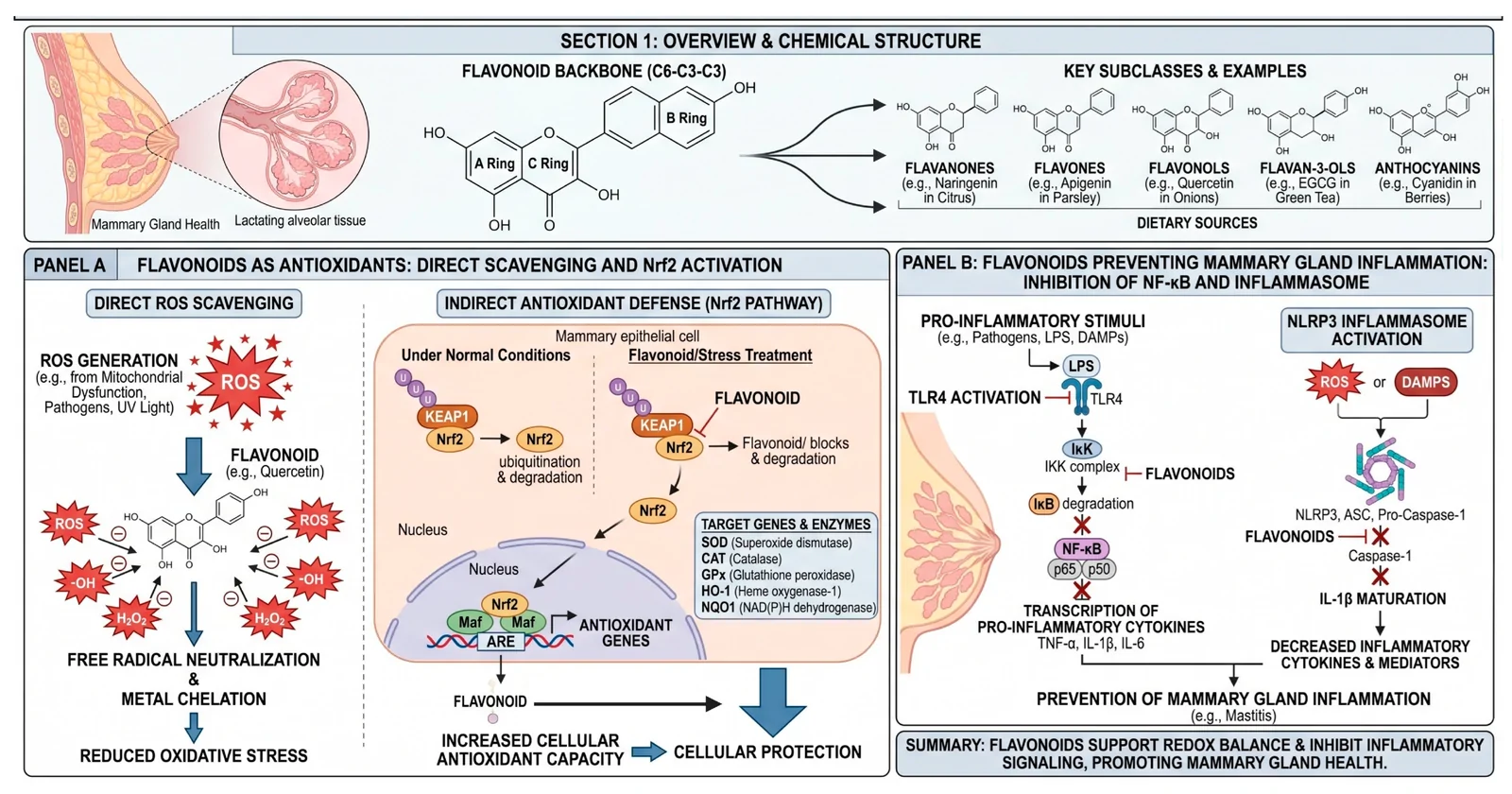
Structural classes of dietary flavonoids and their dual antioxidant–anti-inflammatory actions. (Structures) Flavonoids share a C6–C3–C6 backbone; representative subclasses are flavanones (naringenin), flavones (apigenin), flavonols (quercetin), flavan-3-ols (EGCG) and anthocyanins (cyanidin). (Panel A) Antioxidant actions: direct radical scavenging and metal chelation, together with Keap1–Nrf2-driven transcription of cytoprotective enzymes. (Panel B) Anti-inflammatory actions: inhibition of TLR4–IKK–NF-κB signalling, of NLRP3 inflammasome assembly and of downstream cytokine release. ───┤represents inhibition or block.

Oligomeric proanthocyanidins, which are oligomers of catechin and epicatechin units, expand the flavan-3-ol pharmacological profile into a direct bovine mammary context. In LPS-stimulated MAC-T cells, oligomeric proanthocyanidins attenuate pro-inflammatory cytokine production (TNF-α, IL-1β, IL-6) and downregulate COX-2 and iNOS by simultaneously inhibiting NF-κB and MAPK signalling cascades [98]. This dual-pathway blockade in the bovine mammary epithelium itself reinforces the catechin subclass as broadly active across both monomeric and oligomeric chemotypes, and complements the predominantly hepatic and rodent-mammary evidence that dominates the EGCG literature. An integrated translational model linking intake, bioavailability, systemic mediators and mammary tissue effects across all six subclasses is summarized in Figure 4. 

## 12. Cross-Cutting Themes and Translational Considerations

### 12.1. Mechanistic Convergence and Structural Divergence

Taken together, the mastitis literature shows extensive mechanistic convergence at three core nodes—TLR4/NF-κB, MAPK, and Nrf2/HO-1—and selective divergence at less-canonical targets. Dual NF-κB plus MAPK inhibition is shared by structurally unrelated compounds (myricetin, luteolin, wogonin, ISL, licochalcone A, EGCG, daidzein, farrerol, oligomeric proanthocyanidins), suggesting that simultaneous engagement of these parallel cascades is a recurrent property of high-potency flavonoid anti-inflammatories [44,45,98]. AKT-proximal inhibition is more selective and is observed with myricetin, licochalcone A, wogonin, and farrerol. NLRP3 inflammasome restraint is concentrated in baicalein, naringenin, and rutin, while ferroptosis inhibition through SIRT1 signaling is currently best evidenced for fisetin, puerarin, and diosmetin [72]. The AhR–Src–NF-κB axis remains uniquely the province of formononetin [48]. This pattern argues against viewing individual flavonoids as interchangeable and instead supports informed compound selection based on the specific pathobiological emphasis of the mastitis context—e.g., periparturient oxidative stress, Gram-positive biofilm-mediated chronicity, or post-partum acute LPS-driven injury.

### 12.2. The Host–Pathogen Interface and Antimicrobial Resistance

A particularly translationally significant theme is the dual host- and pathogen-directed action of several flavonoids. Quercetin glycosides, kaempferol, rutin, and ISL exhibit anti-biofilm activity, with IGPD emerging as a generalizable druggable target across coagulase-negative staphylococci [73,74,77]. Myricetin extends this anti-virulence repertoire to *Pseudomonas aeruginosa* by inhibiting biofilm formation, quorum sensing, and virulence factor production [78]. Baicalein interferes with *S. agalactiae* pbsP-mediated adhesion and core energy metabolism at sub-inhibitory concentrations [76]. EGCG reverses β-lactam resistance in MRSA by perturbing the membrane [94]. In the context of escalating antimicrobial resistance and tightening regulation of intramammary antibiotic use, these mechanisms position flavonoids less as antibiotic substitutes and more as resistance-modifying adjuncts, capable of restoring or potentiating conventional antibacterial activity. The host–pathogen interactions summarized above were characterized primarily in vitro and in bovine, ovine or murine mastitis-relevant systems; because such systems describe host-defense, biofilm and redox/immune signaling principles that underlie infection and antimicrobial-resistance biology in mammals generally, their potential relevance to human antimicrobial stewardship is noted here for context, which is also why human-relevant pathogens such as MRSA are discussed alongside ruminant-specific organisms. Direct clinical confirmation of these resistance-modifying mechanisms remains limited in both ruminants and humans at present, however, and dedicated, separately designed studies in each species will be needed before any such parallel can be considered established evidence for human antimicrobial-resistance management.

### 12.3. The Gut–Mammary Axis

FMT-validated evidence across hesperetin, neohesperidin, naringenin, and fisetin [13,46,62,63] establishes that flavonoid-induced gut microbiota remodeling can be causally responsible for mammary protection. The recurrence of this mechanism across structurally related flavanones—extended further by the systemic metabolic and oxidative effects of dietary naringin in transition cows [88]—suggests it is a class-level property rather than a compound-specific idiosyncrasy. Two implications follow. First, fasting microbiome composition is likely to be an important determinant of inter-individual variability in flavonoid efficacy, supporting microbiota-stratified dosing strategies. Second, germ-free animal studies and targeted microbiome-depletion designs are needed to formally distinguish microbiota-dependent from microbiota-independent contributions to in vivo efficacy. All FMT-validation data underpinning the gut–mammary axis described in this section derive from murine donor–recipient experiments; because the gut-microbiota and mucosal immune mechanisms captured in these murine models reflect principles that are broadly conserved across mammals, including both ruminants and humans, this evidence base is also the reason a parallel gut–mammary relationship in human lactation is plausible and is noted here for context. Ruminant gut physiology and rumen-dependent metabolism nonetheless differ substantially from those of monogastric species, and direct confirmatory evidence of a flavonoid-modulated gut–mammary axis has not yet been established in either dairy cattle, sheep, goats or humans; formally testing and comparing this relationship across species, rather than assuming equivalence, remains a priority for future research.

### 12.4. Bioavailability, Formulation, and Translational Gaps

Most flavonoids exhibit well-documented bioavailability limitations due to extensive phase II metabolism, low aqueous solubility, and rapid efflux. The daidzin–daidzein contrast [67] illustrates how deglycosylation can be a prerequisite for full pharmacological activity, while puerarin’s C-glycosidic bond resists deglycosylation and may shift its absorption profile [70]. Concentration-dependent profiling of hesperetin and naringenin further indicates that the dominant pharmacological action—antioxidant, anti-inflammatory, or directly antibacterial—shifts with achievable plasma and tissue concentrations [86], reinforcing the need for formulations that achieve target-compartment exposures. The [87] pilot study in mastitic Holstein–Friesian cows—demonstrating intramammary tolerability and somatic cell count reduction by hesperidin, naringenin, and chrysin—remains a rare clinical anchor. It should be emphasized that, with this and a small number of other exceptions, the mechanistic claims summarized throughout this review are derived predominantly from in vitro cell-culture assays and murine mastitis models; such systems are valuable for hypothesis generation and mechanistic dissection but do not reproduce the dose, pharmacokinetics, immune complexity or udder anatomy of the lactating ruminant mammary gland, and the magnitude, and in some cases the direction, of effects observed in vitro may not translate directly to in vivo or field conditions. Statements of efficacy in this review should accordingly be read as mechanistically supported hypotheses awaiting confirmation in appropriately powered ruminant studies, rather than as established clinical findings. Translational priorities should therefore include (i) controlled efficacy trials with microbiological outcomes in large-animal cohorts, (ii) intramammary delivery formulations that bypass first-pass metabolism, (iii) compound–pathogen pairing strategies (e.g., baicalin/baicalein for *S. agalactiae*, ISL or rutin for *S. xylosus*, myricetin for *P. aeruginosa*, EGCG-based combinations for MRSA), and (iv) systematic assessment of compound combinations targeting complementary mechanisms (e.g., EGCG plus hydroxytyrosol). A compound-level summary of the distinctive mechanisms, principal experimental models and key references for each flavonoid discussed in this review is provided in Table 2.

### 12.5. Factors Influencing the Bioavailability and Bioactivity of Flavonoids

The in vivo efficacy of any flavonoid is ultimately constrained by its bioavailability, which is governed first by chemical structure. Aglycones are generally more membrane-permeable than their corresponding glycosides, and the daidzin–daidzein contrast illustrates that intestinal deglycosylation can be a prerequisite for full pharmacological activity [61]. The nature of the glycosidic bond is equally important: O-glycosides are hydrolyzed by lactase-phlorizin hydrolase and cytosolic β-glucosidase in the small intestine, whereas C-glycosides such as puerarin resist deglycosylation and are absorbed by a different route, altering their pharmacokinetic profile [70]. The degrees of hydroxylation, methylation, glycosylation, and overall molecular size further determine lipophilicity, aqueous solubility, and membrane permeability, and hence the fraction of an ingested dose that reaches the circulation.

Once absorbed, most flavonoids undergo extensive phase II metabolism—glucuronidation, sulfation and methylation in the intestinal wall and liver—so the species circulating in plasma are predominantly conjugates whose bioactivity may differ markedly from that of the parent compound; low aqueous solubility and active efflux by membrane transporters further limit systemic exposure. The gut microbiota is a decisive and often underappreciated determinant of flavonoid action: bacterial ring fission generates smaller, sometimes more bioactive, phenolic metabolites, and interindividual differences in microbial composition give rise to distinct metabotypes. This is exemplified by the division of isoflavone consumers into equol producers and non-producers and by the enhanced bioactivity of colonic epicatechin metabolites relative to the parent molecule [97]. Such microbiota-dependent bioactivation parallels the gut–mammary mechanisms described above and is a major source of the variability seen in clinical responses to flavonoid supplementation.

The dominant pharmacological action of a flavonoid is itself concentration-dependent: hesperetin and naringenin shift from predominantly antioxidant and anti-inflammatory effects at lower concentrations to direct antibacterial activity at higher concentrations, so the exposure actually achieved in the target tissue dictates which mechanism prevails [86]. Host and species factors add further complexity—ruminal microbial metabolism in dairy ruminants, physiological state during the periparturient window, age, diet, and genetic variation in metabolizing enzymes all modulate the absorbed dose and its fate—and caution strongly against the uncritical extrapolation of in vitro potency to the lactating animal. These limitations motivate formulation and delivery strategies designed to raise and sustain target-compartment exposure, including nanoencapsulation, phospholipid (phytosome) complexes, liposomes and micellar carriers, and, specifically for the mammary gland, intramammary administration that bypasses first-pass metabolism. The pilot demonstration that intramammary hesperidin, naringenin and chrysin are well tolerated and reduce milk somatic cell count provides an early proof of concept for this last approach [86], but systematic pharmacokinetic and bioavailability studies in dairy ruminants remain a clear priority for translation.

### 12.6. Critical Comparison Across Flavonoid Subclasses: Strength of Evidence, Mechanistic Consistency and Translational Readiness

Section 12.1 identified the pathways on which the six subclasses converge. A complementary—and more translationally consequential—question is which subclasses are currently best supported by evidence. When the subclasses are ranked by the highest level of evidence attained, rather than by publication volume, the resulting order differs substantially from what the size of the literature alone would suggest. Flavanones are currently the best supported (Table 3): they are the only subclass with a clinical demonstration in lactating dairy cattle, where intramammary administration of hesperidin, naringenin, and chrysin reduced milk somatic cell count without adverse hematological or biochemical effects [87]—and the only subclass in which a proposed mechanism, gut microbiota remodeling, has been tested causally rather than inferred, through fecal microbiota transplantation across three independent compounds [51,52,53]. Flavones are supported by the single most rigorous study in this literature: baicalin was evaluated in two species in vivo, in mice and in intramammary-challenged Holstein cows, with its proposed target validated genetically by phenocopying in IL-17RA-knockout mice [85]. Flavonols provide the largest and most mechanistically richest body of work and the only ruminant feeding trial with redox endpoints (rutin in transition Hu sheep [33,69], yet no flavonol has been tested for efficacy against mastitis in cattle. Isoflavones occupy an intermediate position: supplementation data exist in mastitic and heat-stressed dairy cows [70,90], but endocrine-safety questions remain unaddressed. Chalcones and flavan-3-ols, despite considerable mechanistic precision—quantified IGPD binding for isoliquiritigenin [74] and phenotypic reversal of β-lactam resistance in MRSA for EGCG [94]—have no in vivo ruminant data at all.

Judged by consistency rather than novelty, three pathways are engaged by every subclass reviewed: TLR4/TLR2–MyD88–NF-κB, the p38/ERK/JNK MAPK cascades, and Keap1–Nrf2–ARE/HO-1. Two further nodes recur across several but not all subclasses: tight-junction restoration of the blood–milk barrier (flavonols, flavanones, isoflavones, chalcones) and NLRP3 inflammasome restraint (flavonols, flavones, flavanones). The remaining mechanisms are each confined to one or two compounds—m^6^A/YTHDF2 regulation to quercetin [61], IL-17RA engagement to baicalin [85], the AhR–Src axis to formononetin [48]—and, however mechanistically interesting, should be treated as single-study observations pending independent replication. The practical implication is that the three convergent pathways offer the most dependable basis for compound selection, whereas the divergent mechanisms are best regarded as hypotheses for targeted development rather than as established points of differentiation.

Combining these two assessments, three development priorities emerge. Flavanones are the most defensible candidates for intramammary formulation, because both a tolerability signal and a causally validated systemic mechanism already exist. Baicalin is the compound closest to a confirmatory multi-herd efficacy trial, having already been examined in cattle with a genetically validated target. The anti-virulence and resistance-modifying compounds—isoliquiritigenin, rutin, myricetin, baicalein and EGCG—are best positioned as adjuncts intended to potentiate conventional therapy rather than as antibiotic replacements, since none has demonstrated bacteriological cure as a monotherapy. Three gaps are common to all six subclasses: the absence of dose–response data in lactating ruminants, the absence of bacteriological cure endpoints, and the near-complete absence of independent replication, with most compounds supported by findings from a single research group. Table 3 summarizes this comparison; the conflicting findings and implementation barriers that underlie these gaps are examined in Section 12.7.

### 12.7. Conflicting Findings, Limitations of the Current Evidence Base, and Challenges to Clinical Implementation

The literature synthesized above is not internally consistent, and several tensions merit explicit acknowledgment. The clearest concerns the relationship between glycosylation and activity. The daidzin–daidzein comparison supports a general rule that aglycones are pharmacologically superior because of greater membrane permeability [67], yet two of the strongest in vivo datasets in this review contradict it. Baicalin, a glucuronide, was effective after oral and systemic administration in both mice and Holstein cows [85], whereas its aglycone baicalein has been characterized almost exclusively in vitro [76], and in the only clinical study in lactating cattle, it was the glycoside hesperidin rather than the aglycone hesperetin that reduced somatic cell count [87]. Glycosylation evidently alters absorption route, plasma half-life and tissue distribution in ways that cannot be reduced to a simple aglycone-superiority rule, and formulation decisions taken on that basis alone would be premature.

A second and more consequential inconsistency concerns the desired direction of the immunological effect. Mixed soybean isoflavones enriched Fc-gamma-receptor-mediated phagocytosis and complement pathways in bovine mammary epithelial cells and restrained *S. agalactiae* internalization [89], whereas genistein and equol suppressed IL-12/IL-18-driven IFN-γ production by NK cells [91]. Both outcomes are presented as beneficial, yet they are immunologically opposite, and the latter raises the possibility that potent immunomodulation could impair rather than assist bacterial clearance. The existing literature cannot resolve this, because the great majority of in vivo studies reviewed here used sterile LPS or LTA challenge, in which no viable pathogen is present, and clearance cannot be measured; among the minority employing live-pathogen models [49,64,72,85], bacterial load is seldom reported as a primary endpoint. Until intramammary bacterial counts and cure rates are measured alongside inflammatory markers, the assumption that flavonoids dampen inflammation without compromising host defense remains untested.

A third inconsistency concerns the strength of evidence for direct molecular targets. Where binding affinity has been measured, it is modest: isoliquiritigenin binds IGPD with a K_D of 234 µM [74], several orders of magnitude weaker than would be expected of a lead compound, suggesting that IGPD inhibition contributes to rather than accounts for the observed anti-biofilm effect. The naringenin–IκBα interaction rests on molecular docking alone [46] and has not been corroborated by biophysical binding measurement, mutagenesis, or structural analysis. Anti-virulence effects described as “sub-inhibitory” concentrations are likewise difficult to interpret when the corresponding MIC exceeds 1024 µg/mL, as for baicalein against *S. agalactiae* [76], because a concentration below such an MIC may nonetheless lie far above anything attainable in milk or mammary tissue.

Beyond these specific conflicts, four features constrain the evidence base as a whole. First, model dependence: most mechanistic findings derive from immortalized cell lines (MAC-T, EpH4-Ev, RAW264.7) and murine mastitis models that differ from the lactating ruminant udder in anatomy, milk-fraction pharmacokinetics, resident immune cell composition and metabolic load (Section 12.4). Second, study design: most in vivo protocols administer the flavonoid before challenge—naringenin, for instance, for seven days prior to intramammary LPS [46]—and therefore test prophylaxis rather than treatment of established disease, which is the clinically relevant scenario. Third, route and dose: intraperitoneal administration is common in murine work [65] but has no counterpart in dairy practice, and doses are rarely scaled to achievable dietary or intramammary exposures. Fourth, reporting: the literature is almost uniformly positive, head-to-head comparisons between compounds are essentially absent, null findings are seldom published, and dose, purity, vehicle (commonly DMSO) and challenge intensity vary so widely between studies that quantitative cross-compound comparison—and hence any evidence-based ranking of potency—is not presently possible.

Clinical implementation faces further, largely unexamined obstacles. In ruminants, orally administered flavonoids undergo extensive ruminal microbial metabolism, including deglycosylation and C-ring fission, before absorption; the metabolite profile reaching the mammary gland is therefore unlikely to resemble that measured in monogastric species, and rumen-protected or intramammary delivery will be necessary wherever the parent structure is the active species. No study cited in this review reports flavonoid or metabolite concentrations in the milk or mammary tissue of dairy ruminants, so it is not known whether concentrations effective in vitro are attainable in vivo at any practical dose. Milk residues, withdrawal periods and effects on organoleptic and processing characteristics—plausible in particular for astringent, protein-binding catechins—have not been investigated. Regulatory status is unsettled, as flavonoids are variously classified as feed additives, nutraceuticals or veterinary medicinal products depending on jurisdiction and route, and intramammary administration would require formal authorization supported by residue and safety data that do not currently exist. Species-specific safety questions also persist, most notably the recognized phytoestrogenic effects of isoflavones such as formononetin on ovine reproduction, which none of the mastitis studies cited here has evaluated. Finally, cost of goods, stability during feed processing and storage, and the practicality of repeated intramammary dosing within a commercial milking routine will ultimately determine whether mechanistically attractive compounds are adoptable on farms.

Resolving these issues will require a change in study design rather than further descriptive mechanistic work: randomized, blinded and adequately powered trials in lactating ruminants using live-pathogen challenge or naturally occurring infection; bacteriological cure and somatic cell count as co-primary endpoints alongside inflammatory markers; treatment rather than pretreatment protocols; pharmacokinetic characterization in milk and mammary tissue; and pre-specified reporting of null results. Until such studies exist, the conclusions of this review—and of the field more broadly—are best read as a mechanistically coherent and biologically plausible rationale for the investigation of flavonoids in mastitis, rather than as evidence that any individual compound is clinically effective.

## 13. Conclusions

Across the six flavonoid subclasses considered here, a coherent therapeutic logic for mammary inflammation emerges. Structurally diverse compounds converge on a small set of master regulatory nodes—TLR4/NF-κB, MAPK, and Keap1/Nrf2/HO-1—that together control the inflammatory–oxidative axis underlying mastitis. Around this shared core, individual flavonoids contribute distinguishing actions: epitranscriptomic regulation by quercetin, IL-17RA targeting by baicalin, anti-virulence engagement of bacterial IGPD by rutin and isoliquiritigenin, AhR-driven Src/NF-κB blockade by formononetin, SIRT1-dependent ferroptosis inhibition by puerarin and fisetin, gut–mammary axis remodeling across the flavanones, and resistance-reversing membrane perturbation by EGCG against MRSA.

This multi-target architecture is well-matched to the polymicrobial, redox-driven, and barrier-disruptive nature of clinical mastitis and offers credible mechanistic alternatives or adjuncts to antibiotic-centric strategies. Translational maturation will require systematic large-animal efficacy trials with microbiological endpoints, bioavailability-optimized delivery—particularly intramammary formulations—microbiota-stratified dosing strategies, and rational compound combinations selected based on complementary mechanisms rather than empirical mixing. It must be emphasized, however, that the evidence is presently unbalanced: flavanones and the flavone baicalin are supported by data in cattle, whereas chalcones and flavan-3-ols have not been examined in any ruminant in vivo, and no compound in any subclass has yet been shown to improve bacteriological cure. The conflicting findings on glycoside versus aglycone activity, the untested effect of anti-inflammatory action on bacterial clearance, and the absence of pharmacokinetic, residue and regulatory data in lactating ruminants define the agenda that must be completed before clinical recommendations can be made. Within a redox biology framework, flavonoids are best understood not as single-target anti-inflammatories but as upstream interruptors of the self-amplifying inflammatory–oxidative loop that defines mammary disease, and as such warrant continued, mechanistically rigorous translational development.

## Figures and Tables

**Table 1:** Major molecular pathways modulated by flavonoids in mammary gland inflammation.

Pathway/Target	Mechanistic Role and Flavonoid Effect	Representative Flavonoids	Principal Experimental Model(s)	References
TLR4–MyD88–IKK–NF-κB	Canonical PAMP-sensing axis. Flavonoids reduce TLR4/CD14/MD2/MyD88 expression, block IKK and IκBα phosphorylation and prevent p65 nuclear translocation.	Quercetin, myricetin, kaempferol, luteolin, apigenin, hesperetin, naringenin, ISL	LPS-challenged BMECs; LPS-induced murine mastitis	[5,15,42,48,62,64,65]
p38/ERK/JNK MAPK	Parallel inflammatory amplification. Flavonoids attenuate phosphorylation of one or more MAPK arms, dampening AP-1 transcriptional output.	Licochalcone A, myricetin, luteolin, ISL, EGCG, daidzein	LPS-induced murine mastitis; BMECs/mMECs	[22,43,54,66,67,68]
Keap1–Nrf2–ARE/HO-1	Cytoprotective transcriptional programme. Flavonoids modify Keap1 cysteines, releasing Nrf2 to drive antioxidant gene expression (HO-1, NQO1, SOD, GPX4).	Myricetin, rutin, fisetin, baicalin, neohesperidin, puerarin, EGCG	H_2_O_2_-challenged BMECs; LPS-induced murine mastitis	[13,44,50,59,60,69,70]
NLRP3 inflammasome/caspase-1	Maturation of IL-1β and IL-18. Flavonoids inhibit assembly and caspase-1 activation, additionally restraining GSDMD-mediated pyroptosis.	Baicalein, naringenin, rutin	LPS-treated BMECs; LPS-induced murine mastitis	[27,28,29,30,31,71]
COX-2/iNOS	Effector enzymes generating prostaglandins and NO. Flavonoids suppress both expression and, for some, direct enzymatic activity.	Quercetin, luteolin, ISL, daidzein	LPS-induced rat and murine mastitis; MAC-T cells	[16,53,54,67]
Tight junctions/blood–milk barrier	ZO-1, occludin, claudin-3/4 restoration; reversal of LTA- and LPS-driven permeabilization.	Quercetin, myricetin, fisetin, neohesperidin, naringenin, licochalcone A	LTA/LPS-challenged MAC-T cells and murine mammary tissue	[43,47,52,62,63,70]
AMPK/mTOR/SIRT1	Energy-sensing and deacetylation control of autophagy, antioxidant defence and inflammasome activity.	Quercetin, myricetin, rutin, puerarin	BMECs; murine mastitis; transition Hu sheep	[47,49,60,69,71]
m^6^A epitranscriptomic regulation	YTHDF2-dependent post-transcriptional control of pro-inflammatory chemokines (e.g., CCL5).	Quercetin	*S. aureus*-induced bovine mastitis (BMECs)	[61]
Ferroptosis (GPX4/SLC7A11)	Iron-dependent regulated cell death restrained through Nrf2/GPX4/xCT and SIRT1/p53/SLC7A11 axes.	Fisetin, puerarin	LPS/*S. aureus*-induced murine mastitis; BMECs	[13,49,72]
AhR–Src–NF-κB	Receptor-mediated suppression of Src kinase, reducing NF-κB activation and preserving barrier integrity.	Formononetin	LPS-induced murine mastitis; EpH4-Ev cells	[48]
Bacterial virulence (IGPD, pbsP, biofilm)	Direct anti-virulence and anti-biofilm effects via inhibition of histidine biosynthesis or adhesion factors.	Quercetin glycosides, rutin, ISL, baicalein, kaempferol, myricetin	In vitro antibacterial/biofilm assays (*S. xylosus*, *S. agalactiae*, *S. aureus*)	[73,74,75,76,77,78]
Gut–mammary axis	FMT-validated remodelling of gut microbiota with downstream mammary protection.	Fisetin, hesperetin, neohesperidin, naringenin	FMT-validated murine mastitis models	[13,46,62,63]

**Table 2:** Compound-level summary of flavonoids with anti-mastitis activity.

Compound	Subclass	Distinctive Mechanism(s) in Mastitis	Principal Model(s)	Evidence Level	References
Quercetin	Flavonol	TLR4/MyD88/NF-κB blockade; AMPK/mTOR autophagy normalization; m^6^A/YTHDF2 regulation of CCL5; anti-biofilm via quercetin-3-O-rhamnoside	LPS-BMECs; LTA MAC-T cells; *S. aureus* murine mastitis	Preclinical (in vitro + murine)	[15,47,61,73,79]
Myricetin	Flavonol	AKT/IKK/NF-κB and p38/ERK inhibition; AMPK/Nrf2-mediated cytoprotection; anti-biofilm/quorum-sensing against *P. aeruginosa*	LPS mouse mastitis; H_2_O_2_-BMECs; *P. aeruginosa*	Preclinical (in vitro + murine)	[42,60,78,81]
Kaempferol	Flavonol	NF-κB p65/IκBα blockade; suppression of ANGPTL2; inhibition of *S. aureus* primary-attachment biofilm phase	LPS murine mastitis; primary MMECs; *S. aureus* biofilm	Preclinical (in vitro + murine)	[65,77,82]
Rutin	Flavonol	NF-κB/GRP78 modulation; SIRT1-dependent NLRP3 and GSDMD suppression; AMPK/Nrf2 in transition sheep; IGPD-targeted anti-biofilm against S. xylosus	LPS murine mastitis; BMECs; Hu sheep; S. xylosus murine model	Preclinical, with emerging ruminant (ovine) data	[33,69,71,75,83]
Fisetin	Flavonol	Nrf2/Keap1/HO-1 and GPX4/xCT-mediated ferroptosis suppression; gut–mammary axis remodelling with SCFA elevation	LPS MAC-T cells; LPS murine mastitis	Preclinical (in vitro + murine)	[13]
Luteolin	Flavone	TLR2/TLR4 down-regulation; NF-κB and p38/ERK/JNK inhibition; MMP-2/9 suppression with TIMP-1/2 induction	*S. aureus* murine mastitis; BMECs	Preclinical (in vitro + murine)	[64,66]
Apigenin	Flavone	TLR4/NF-κB blockade validated by BAY 11-7028; reduction in MPO and cytokines	Intra-nipple LPS rat mastitis	Preclinical (rodent in vivo)	[5]
Baicalin	Flavone	IL-17RA-targeted blockade of MAPK/ERK/NF-κB validated in mice and Holstein cows; Nrf2-dependent cytoprotection; ovine lactational gene induction	Dual-species E. coli mastitis; H_2_O_2_-BMECs; OMECs	Preclinical + bovine/ovine translational data	[51,59,85]
Baicalein	Flavone	Sub-MIC anti-virulence against S. agalactiae; ↓pbsP; suppressed bacterial energy metabolism; host Nrf2 activation	S. agalactiae BMEC invasion	Preclinical (in vitro)	[76]
Wogonin	Flavone	Inhibition of Akt/NF-κB; activation of Nrf2/HO-1 signalling; reduced cytokine output and oxidative damage	LPS murine mastitis; mammary epithelial cells	Preclinical (in vitro + murine)	[44]
Diosmetin	Flavone	Inhibition of SIRT1/GPX4-mediated ferroptosis; reduced lipid peroxidation and iron accumulation	*S. aureus* murine mastitis; BMECs	Preclinical (in vitro + murine)	[72]
Hesperetin/hesperidin	Flavanone	TLR4/NF-κB inhibition; tight-junction restoration; FMT-validated gut–mammary axis effect; SCC reduction in clinical bovine mastitis	LPS murine mastitis; mMECs; clinical Holstein–Friesian cows	Preclinical + bovine clinical (pilot trial)	[62,86,87]
Neohesperidin	Flavanone	TLR4/NF-κB blockade + AMPK/Nrf2/HO-1 induction; FMT-validated gut microbiota remodelling	LPS murine mastitis	Preclinical (murine)	[63]
Naringenin/naringin	Flavanone	Direct IκBα binding (−24.05 kcal/mol) blocking phosphorylation; gut microbiota normalization; systemic metabolic and antioxidant benefit in transition cows	LPS bovine/murine MECs; murine mastitis; transition dairy cows	Preclinical + bovine field data	[46,86,88]
Farrerol	Flavanone (dihydroflavone)	Inhibition of Akt/NF-κB p65, Erk1/2 and p38 signalling; reduced MPO and cytokine output	LPS murine mastitis	Preclinical (murine)	[45]
Daidzein/daidzin	Isoflavone	Aglycone-superior IKK–IκBα–p65 inhibition; MAPK suppression; mammary MAPK/NF-κB blockade	LPS RAW264.7; mouse MECs; lactating dairy cows under heat stress	Preclinical + bovine field data	[22,67,90]
Genistein/equol	Isoflavone	NK-cell IFN-γ restraint via MAPK and IL-18Rα; equol microbiota-dependent	PBMC; soy-fed C57BL/6 mice	Preclinical (in vitro/murine; non-mastitis immune model)	[91]
Soybean isoflavone mix	Isoflavone	Proteomic remodelling of bMEC defence; ↓integrin β5/osteopontin; ↑p27kip1/PIK3CA/CD82; restraint of S. agalactiae invasion	*S. agalactiae*-bMECs	Preclinical (in vitro, bovine cells)	[89]
Formononetin	Isoflavone	AhR-driven Src kinase inactivation and downstream NF-κB blockade; CH223191-reversible	LPS murine mastitis; EpH4-Ev cells	Preclinical (in vitro + murine)	[48]
Puerarin	Isoflavone	Nrf2/HO-1/xCT antioxidant induction; SIRT1/p53/SLC7A11-mediated ferroptosis suppression; in vivo cytokine reduction in dairy cattle	H_2_O_2_-BMECs; mastitic dairy cows; *S. aureus* murine mastitis	Preclinical + bovine clinical data	[49,70]
Isoliquiritigenin	Chalcone	IGPD-targeted anti-biofilm against S. xylosus (K_D = 234 µM); dual NF-κB/MAPK blockade; ↓COX-2/iNOS; systemic antioxidant action	*S. xylosus* murine mastitis; LPS MAC-T cells; LPS cognitive model	Preclinical (in vitro + murine)	[54,74,92]
Licochalcone A	Chalcone	MAPK and AKT/NF-κB blockade; ZO-1/occludin/claudin-3 restoration	LPS murine mastitis; mMECs	Preclinical (in vitro + murine)	[43]
EGCG	Flavan-3-ol	Nrf2-dependent cytoprotection; NF-κB/MAPK/HIF-1α suppression; phenotypic reversal of MRSA β-lactam resistance	H_2_O_2_-BMECs; LPS rat mastitis; MRSA	Preclinical (in vitro + rodent)	[50,68,93,94,95,96]
Epicatechin	Flavan-3-ol	NF-κB/MAPK/JAK-STAT inhibition; Nrf2 induction; microbiota-dependent metabolite bioactivation	Reviewed mechanisms	Preclinical (mechanistic, literature-derived)	[97]
Oligomeric proanthocyanidins	Flavan-3-ol (oligomer)	Dual NF-κB and MAPK suppression; reduced TNF-α/IL-1β/IL-6, COX-2 and iNOS in bovine mammary epithelium	LPS-stimulated MAC-T cells	Preclinical (in vitro, bovine cells)	[98]

**Table 3:** Comparative appraisal of the six flavonoid subclasses by strength of evidence, mechanistic consistency and translational readiness in mammary gland inflammation.

Subclass	Best-Evidenced Compounds	Consistently Modulated Pathways	Highest Level of Evidence Attained	Principal Gaps and Caveats
Flavanones	Hesperetin/hesperidin, naringenin, neohesperidin	TLR4–NF-κB; Nrf2/HO-1; tight junctions; gut–mammary axis	Pilot clinical study in lactating dairy cows (intramammary; SCC endpoint) [87]; FMT causal validation across three compounds [46,62,99]; transition-cow feeding trial [88]	n = 12 in the only clinical study with no bacteriological cure endpoint and no reported blinding, placebo arm, pharmacokinetics or withdrawal data; all FMT evidence is murine
Flavones	Baicalin; baicalein, luteolin, apigenin, wogonin, diosmetin	TLR2/TLR4–NF-κB; MAPK; Nrf2/HO-1; MMP/TIMP balance	Dual-species in vivo challenge (mice and Holstein cows) with genetic target validation in IL-17RA-knockout mice [85]	Baicalin findings not yet independently replicated; baicalein anti-virulence effects are reported at “sub-inhibitory” concentrations, while the MIC exceeds 1024 µg/mL [76], leaving the therapeutic window undefined
Flavonols	Quercetin, rutin, myricetin, fisetin, kaempferol	TLR4/MyD88–NF-κB; MAPK; Nrf2/HO-1; plus m^6^A, ferroptosis and IGPD-directed anti-biofilm activity	Ovine in vivo feeding trial with redox and cytoprotection endpoints (rutin, transition Hu sheep) [33,69]	No efficacy trial in cattle; quercetin oral bioavailability is low; antibacterial activity is reported at 0.07–1.04 mg/mL [73], far above concentrations plausibly attainable in mammary tissue
Isoflavones	Puerarin, daidzein, formononetin	NF-κB; MAPK; Nrf2/HO-1/xCT; SIRT1-dependent ferroptosis control	Supplementation studies in mastitic and in heat-stressed dairy cows [70,90]	Phytoestrogenic activity raises reproductive-safety questions in ruminants that no mastitis study has addressed; equol formation is metabotype-dependent, generating inter-animal variability [91]
Chalcones	Isoliquiritigenin, licochalcone A	NF-κB; MAPK; AKT; COX-2/iNOS; IGPD-directed anti-biofilm activity	Murine mastitis models with quantified target binding (K_D = 234 µM) [74]	No ruminant data of any kind; the α,β-unsaturated carbonyl confers broad thiol reactivity, so target selectivity and safety margins are undefined; the measured affinity is weak for a lead compound
Flavan-3-ols	EGCG, oligomeric proanthocyanidins, epicatechin	Nrf2/HO-1; NF-κB; MAPK; JAK/STAT (epicatechin)	Bovine mammary epithelial (MAC-T) data [98] and phenotypic reversal of MRSA β-lactam resistance in vitro [94]	No in vivo ruminant study; EGCG has the lowest oral bioavailability and greatest chemical instability of the six subclasses; epicatechin activity depends on microbial metabolites and therefore on host microbiota [97]

## Data Availability

No new data were generated for this review article.

## Abbreviations

AhR	Aryl hydrocarbon receptor
AMPK	AMP-activated protein kinase
ARE	Antioxidant response element
BMEC	Bovine mammary epithelial cell
COX-2	Cyclooxygenase-2
EGCG	Epigallocatechin-3-gallate
FMT	Fecal microbiota transplantation
GPX4	Glutathione peroxidase 4
HO-1	Heme oxygenase-1
IGPD	Imidazole glycerol phosphate dehydratase
IKK	IκB kinase
iNOS	Inducible nitric oxide synthase
Keap1	Kelch-like ECH-associated protein 1
LPS	Lipopolysaccharide
LTA	Lipoteichoic acid
MAPK	Mitogen-activated protein kinase
MEC	Mammary epithelial cell
mMEC	Murine mammary epithelial cell
MIC	Minimum inhibitory concentration
MRSA	Methicillin-resistant *Staphylococcus aureus*
NF-κB	Nuclear factor kappa B
NLRP3	NOD-like receptor family pyrin domain-containing protein 3
Nrf2	Nuclear factor erythroid 2-related factor 2
OMEC	Ovine mammary epithelial cell
PAMP	Pathogen-associated molecular pattern
ROS	Reactive oxygen species
SCFA	Short-chain fatty acid
SOD	Superoxide dismutase
TLR	Toll-like receptor
ZO-1	Zonula occludens-1
